# Resilience and the reduction of occupational stress in Nursing

**DOI:** 10.1590/1518-8345.5866.3636

**Published:** 2022-10-07

**Authors:** Carmen Cristiane Schultz, Christiane de Fátima Colet, Eliane Raquel Rieth Benetti, Juliana Petri Tavares, Eniva Miladi Fernandes Stumm, Patrícia Treviso

**Affiliations:** 1 Universidade Regional do Noroeste do Estado do Rio Grande do Sul, Ijuí, RS, Brazil.; 2 Universidade Federal de Santa Maria, Hospital Universitário de Santa Maria, Santa Maria, RS, Brazil.; 3 Universidade Federal do Rio Grande do Sul, Porto Alegre, RS, Brazil.; 4 Universidade do Vale do Rio dos Sinos (UNISINOS), Porto Alegre, RS, Brazil.

**Keywords:** Occupational Stress, Resilience, Psychological, Nursing, Hospital Care, Occupational Health, Occupational Risks, Estresse Ocupacional, Resiliência Psicológica, Enfermagem, Assistência Hospitalar, Saúde do Trabalhador, Riscos Ocupacionais, Estrés Laboral, Resiliencia Psicológica, Enfermería, Atención Hospitalaria, Salud Laboral, Riesgos Laborales

## Abstract

**Objective:**

to analyze the association between resilience and occupational stress of Nursing professionals from a general hospital.

**Method:**

an observational, cross-sectional study involving 321 Nursing professionals. The data collected were: socio-demographic and labour variables, stress and resilience, analyzed with descriptive and inferential statistics.

**Results:**

54.5% of the participants presented moderate resilience and 36.4%, high; 73.5% were at risk of exposure to occupational stress; the relationship between psychological demands and professional category (p=0.009), between control over work and age (p=0.04), professional category (p<0.001), having a management position (p=0.009), being a specialist (p=0.006) and between social support and professional category (p<0.001), having a management position (p=0.03), daily working hours (p=0.03), being a specialist (p<0.001) were verified. There was an association between resilience Factor I - resolutions of actions and values and control over work (p=0.04) and social support (p=0.002).

**Conclusion:**

the Nursing professionals of a general hospital have moderate to high resilience which, associated with high control over their work and high social support, may contribute to the reduction of exposure to occupational stress.

## Resumo

**Objetivo:**

analisar a associação entre resiliência e estresse ocupacional de profissionais de Enfermagem de um hospital geral.

**Método:**

estudo observacional, transversal, envolvendo 321 profissionais de Enfermagem. Os dados coletados foram: variáveis sociodemográficas e laborais, estresse e resiliência, analisadas com estatística descritiva e inferencial.

**Resultados:**

54,5% dos participantes apresentaram resiliência moderada e 36,4%, alta; 73,5% estavam em risco de exposição ao estresse ocupacional; verificada a relação entre demandas psicológicas e categoria profissional (p=0,009), entre controle sobre o trabalho e idade (p=0,04), categoria profissional (p<0,001), exercer cargo de chefia (p=0,009), ser especialista (p=0,006) e entre suporte social e categoria profissional (p<0,001), exercer cargo de chefia (p=0,03), jornada diária de trabalho (p=0,03), ser especialista (p<0,001). Houve associação entre o Fator I de resiliência - resoluções de ações e valores e controle sobre o trabalho (p=0,04) e o apoio social (p=0,002).

**Conclusão:**

os profissionais de Enfermagem de um hospital geral apresentaram moderada a alta resiliência que, associada ao alto controle sobre o trabalho e ao elevado apoio social, pode contribuir para a redução da exposição ao estresse ocupacional.

## Resumen

**Objetivo:**

analizar la asociación entre resiliencia y estrés ocupacional de profesionales de Enfermería de un hospital general.

**Método:**

estudio observacional y transversal, realizado en 321 profesionales de Enfermería. Los datos recolectados fueron: variables sociodemográficas y laborales, estrés y resiliencia, analizados con estadística descriptiva e inferencial.

**Resultados:**

54,5% de los participantes presentaron resiliencia moderada y 36,4% alta; 73,5% estaban en riesgo de exposición al estrés ocupacional; fue verificada la relación entre demandas psicológicas y categoría profesional (p=0,009), entre control sobre el trabajo y edad (p=0,04), categoría profesional (p<0,001), ejercer cargo de jefatura (p=0,009), ser especialista (p=0,006) y entre soporte social y categoría profesional (p<0,001), ejercer cargo de jefatura (p=0,03), jornada diaria de trabajo (p=0,03), ser especialista (p<0,001). Se encontró asociación entre el Factor I de resiliencia [resolución de acciones y valores y control sobre el trabajo (p=0,04)] y el apoyo social (p=0,002).

**Conclusión:**

los profesionales de Enfermería de un hospital general presentaron de moderada a alta resiliencia, que asociada al alto control sobre el trabajo y al elevado apoyo social puede contribuir para la reducción de la exposición al estrés ocupacional.

**Highlights**

(1) Resilience, job control and social support can contribute to stress reduction.

(2) Positive correlation between social support and psychological demands in Nursing.

(3) Association between resilience and control over work in Nursing.

(4) Association between resilience and social support in Nursing.

(5) Resilience did not contribute to the reduction of occupational stress in the study population.

## Introduction

Stress has become a common health problem, with significant repercussions in the worker’s life. Psychosocial factors arising from the interaction of the individual with the work environment, its work demands, conditions and organizational structure can influence health and job satisfaction^1^. 

Occupational stress, besides causing impacts on the daily work of nursing, in view of the physical, psychological, social and cultural damage resulting from it, is reflected in the family, in the institution and in society^2^. Characteristics of the nursing work in the hospital context, such as constant exposure to biological, chemical and ergonomic loads, as well as to psychological demands and unfavorable working conditions and the working environment itself, contribute to the worker’s physical and psychological illness^3^.

Factors such as organizational structure, nature and work environment predispose the nursing professional to occupational stress^4^. In addition, the intense pace, the high cognitive and emotional demands, shift work, physical and psychological aggravations^5^, stressful situations, conflicting relationships, pressing risk of errors and losses permeate the day-to-day work and have repercussions on the worker’s mental health, with repercussions on the assistance^6^. 

A study of nurses in Spain affirmed the negative relationship between Nursing occupational stress, the work environment and coping with death^7^. In this sense, a Brazilian investigation evidenced occupational stress, at medium or high level, in 57.4% of the Nursing professionals investigated and explained that the highest levels of stress were associated with the professional category of being a nurse, the shortest time of training, facing the death of the patient and attending to the emergencies and needs of the family members^8^. 

Exposure to stress is influenced by personal and professional characteristics, such as gender, marital status, parenthood, work regime, dual employment status, shift and weekly working hours^2^. A study with Nursing professionals from a university hospital pointed out night work, the simultaneous performance of different tasks combined with frequent interruptions, work overload and the lack of sufficient time to provide care and emotional support to the patient among the main stressors in the profession^4^. 

As for the symptoms resulting from stress, besides physical alterations, psychological alterations can be perceived, such as emotional lability, anxiety, fatigue, among others, which interfere with patient care and professional satisfaction^9^. In this sense, the early identification of the main stressors in the work of nursing enables the development of strategies for the promotion and protection of health and prevention of occupational illness in the context of work organization^10^. The ability to cope with stressors depends on the support offered to the professional and the demands of the context and requires the implementation of intervention programs aimed at promoting coping strategies focused on overcoming vulnerabilities^9^. 

Among the strategies to overcome the difficulties of everyday work in Nursing, studies have focused on resilience^11^, considered a defense mechanism against the threats of suffering or illness, which enables the individual to recover, learn and become stronger to face challenges^12^, constituting an internal reconfiguration that favours positive and creative attitudes and perceptions of the human being when facing difficulties^13^. A study with Nursing professionals, which scored the risk of physical and psychological illness of the category, made explicit the correlation between psychosocial stress and resilience and the need to reorganize work processes and encourage programs that promote resilience in Nursing^14^. 

Identifying factors that contribute to reducing work stress among nursing professionals in the hospital environment and coping strategies can directly impact working conditions and, indirectly, the quality and safety of care provided to patients. Given the above, this study aimed to analyze the association between resilience and occupational stress of Nursing professionals from a general hospital.

## Method

This text has been organized in accordance with the Strengthening the Reporting of Observational Studies in Epidemiology (STROBE): guidelines for reporting observational studies, from the Enhancing the QUAlity and Transparency Of Health Research Network (EQUATOR Network).

### Type of study

This is an observational, exploratory, cross-sectional study. 

### Data collection site

The study was developed in a philanthropic hospital with 225 beds, a macro-regional reference in health, located in a city in the North-western Region of the State of Rio Grande do Sul (RS), Brazil.

### Period

Data collection took place from December 2019 to March 2020.

### Population

The target population of the study comprised 527 Nursing professionals, of whom 90 were nurses and 437 Nursing technicians.

### Selection criteria

The inclusion criteria established were: being a Nursing professional and working in the Nursing service of the institution, regardless of how long they had worked. Five nurses and 59 Nursing technicians who, during data collection, were on vacation, on sick leave, or on maternity leave were excluded; two nurses and ten technicians who did not agree to participate in the study and 130 Nursing technicians who did not respond to the instrument after the third Google Forms^®^ link was sent via WhatsApp^®^, provided by the professional himself. The sample was composed of 321 Nursing professionals, of whom 83 were nurses and 238 Nursing technicians.

### Participants

There was no sample size calculation, since all Nursing professionals of the institution were eligible and were invited to participate in the study. However, from a total of 527 eligible professionals, 321 (60.9%) participated in the study. This quantitative allows us to infer that these data have a confidence level of 99% and sampling error of 3%, which demonstrates the reproducibility of the data collected.

### Study measures

The outcome variable evaluated in this study was exposure to occupational stress. The explanatory variables were resilience and socio-demographic and labour characteristics: gender; age; marital status; category; position held; shift; daily and weekly work hours; time of graduation (years); how long he has worked in Nursing; graduate courses; work unit and presence of employment in another institution.

### Instruments used to collect the information

For data collection, a questionnaire was used for socio-demographic and work characterization, the Job Stress Scale (JSS) and the Resilience Scale (RS). 

The socio-demographic and labour characterization questionnaire was composed of the following variables: gender; age; marital status; category; time of training and work in Nursing; graduate courses; position; shift; daily and weekly work hours; work unit and other employment relationship.

The exposure to occupational stress was evaluated according to the Demand-Control Model (DCM), using the JSS translated and adapted to Portuguese^15^, which evaluates psychosocial factors and exposure to stress in work activities. It is a self-administered scale, with 17 questions on a Likert scale, distributed into three dimensions:1) psychological demand (questions one to five) - assesses the time and speed to perform tasks and the existence of conflict between different demands; 2) control (questions six to 11) - assesses the use and development of skills and authority to make decisions at work and 3) social support (questions 12 to 17) - assesses the worker’s perception of support from managers and colleagues in their work environment^15^. For the demand and control questions, the score ranges from one (never or almost never) to four (often); for the social support questions, the score ranges from four (strongly agree) to one (strongly disagree). Questions four (“Do you have enough time to do all the tasks of your job?”) and nine (“In your job, do you have to repeat the same task many times?”) were reversed to calculate the final score according to the rules of the original instrument. For each dimension of the scale, the higher the score, the greater the psychological demand, the control over work, or the social support perceived by the worker^15^. 

In the bivariate statistical analysis of the JSS, for the dichotomization, due to the lack of data symmetry, the median of the total score of each dimension was used as cut-off point^15^. Values below the median were allocated to the low demand, low control, or low social support groups and values equal to or greater than the median were allocated to the high demand, high control, or high social support groups^15^. The score of the domain “psychological demand” varies from five to 20 points and was dichotomized into low demand (five to 14 points) and high demand (15 to 20 points). The score of the “control over work” dimension ranges from six to 24 points and was dichotomized by the median into low control (nine to 17 points) and high control (18 to 24 points). The score for the “social support” domain ranges from six to 24 points and was dichotomized into low social support (six to ten points) and high social support (11 to 24 points). Finally, the distribution in the quadrants of the DCM^16^ was stratified into low-demand work (high control and low demand), passive work (low control and low demand), active work (high control and high demand) and high-demand work (low control and high demand). According to the theory on which this psychometric instrument is based, the “social support” dimension works as a moderator of work stress^15^.

The RS, developed in 1993^17^ and translated and validated into Portuguese^18^, evaluates the level of positive psychosocial adaptation of the individual in face of life’s striking situations. The instrument includes 25 questions, with response options on a Likert scale ranging from one (strongly disagree) to seven (strongly agree). The sum of the value assigned to each item, at the end, varies between 25 points (less resilience) and 175 points (high resilience)^18^. In this study, we chose to adopt as a classification criterion a score below 121 as low resilience, from 121 to 146 as moderate resilience, and above 147 as high resilience^19^. RS comprises three factors: Factor I represents the sum of the questions characterized by resolutions of actions and values that give meaning to life (1, 2, 6, 8, 10, 12, 14, 16, 18, 19, 21, 23, 24, and 25); Factor II encompasses questions that convey the idea of independence and determination (5, 7, 9, 11, 13, and 22) and Factor III represents the sum of the questions characterized by self-confidence and the ability to adapt to situations (3, 4, 15, 17, and 20)^18^.

### Data collection

For the operationalization of data collection, the Nursing professionals of all shifts and units of the institution were contacted personally, invited to participate and clarified about the objectives and steps of the research. The data were collected, initially, with the use of printed or online forms according to the participant’s choice. Subsequently, due to the pandemic of COVID-19, the respective instruments were sent exclusively online to the participants by Google Forms^®^, via WhatsApp^®^ contact provided by the professional himself, after signing the Free and Informed Consent Form (FICT).

### Data analysis

The data collected on printed forms were typed into Excel^®^ by two independent typists, being compared later, and the returns obtained online were also checked. 

Data was transferred to the Statistical Package for the Social Sciences (SPSS) software, version 22.0, and analyzed with descriptive and inferential statistics. Categorical variables were described by absolute (n) and relative (%) frequencies and quantitative variables by mean, standard deviation (SD) and median. The internal consistency of the scales was analyzed using Cronbach’s alpha coefficient (α), with JSS values of α = 0.677 and RS values of α = 0.905. The Kolmogorov-Smirnov test was used to verify the normality of the variables. Association tests of the variables were employed, among them, the chi-square test, Fisher’s exact test and the Mann-Whitney U test, with p values < 0.05 being considered significant. In Table 1, for the significant analyses, variables with p < 0.05 when associated with the outcome, the odds ratio (OR) was calculated and simple linear regression was performed, considering the Durbin-Watson and the graph of the relationship for the certification of the adequacy to the model.

**Table 1:** Sociodemographic and work characteristics of Nursing professionals (n = 321) working in a general hospital according to the dimensions of the JSS^*^. Ijuí, RS, Brazil, 2019-2020

Variables		n	%	Demand		Control		Social support	
				Low	High	Low	High	Low	High
**Sex**	Female	289	90.0	135(46.7)	154(53.3)	140(48.4)	149(51.6)	142(49.1)	147 (50.9)
	Male	32	10.0	11(34.3)	21(65.7)	10(31.2)	22(68.8)	14(43.7)	18(56.3)
**Age (years)**	18 to 30	106	33.0	51(48.1)	55(51.9)	52(49.1)	54(50.9)	50(47.1)	56(52.9)
	31 to 40	137	42.7	62(45.3)	75(54.7)	71(51.8)	66(48.2)	67(48.9)	70(51.1)
	> 40	78	24.2	33(42.3)	45( 57.7)	27(34.6)	51(65.4)	39(50.0)	39(50.0)
						p = 0.004^†^			
	OR^‡^					1.882 (1.107-3.202)			
	Linear Regression					[F1.390 = 5.59, p=0.019; R^2^=0.017			
**Marital Status**	Married	191	59.5	94(49.2)	97(50.8)	89(46.6)	102(53.4)	92(48.2)	99(51.8)
	Single	130	40.5	52(40.0)	78(60.0)	61(46.9)	69(53.1)	64(49.2)	66(50.8)
**Category**	Nurse	83	25.9	28(33.7)	55(66.3)	25(30.1)	58(69.9)	24(28.9)	59(71.1)
	Technical	238	74.1	118(49.6)	120(50)	125(52.5)	113(47.5)	132(55.4)	106(44.6)
				p = 0.009^§^		p<0.001^§^		p<0.001^§^	
	OR			0.518 (0.307-0.872)		0.390 (0.229- 0.664)		0.327 (0.191-0.560)	
	Linear regression			[F1.390 = 14.21, p=0.000; R^2^=0.043		[F1,390 = 12.82, p=0.000; R^2^=0.039		[F1,390 = 18.23, p=0.000; R^2^=0.054	
**Holds a leadership position**	Yes	34	10.6	16(47.0)	18(53.0)	9(26.5)	25(73.5)	11(32.3)	23(67.7)
	No	287	89.4	130(45.3)	157(54.7)	141(49.1)	146(50.9)	145(50.5)	142(49.5)
						p = 0.009^§^		p = 0.033^§^	
	OR					0.373 (0.168-0.827)		0.468 (0.220-0.998)	
	Linear regression					[F1.390 = 6.355, p=0.012; R^2^=0.020			
**Working hours (hours)**	12	69	21.5	27(39.1)	42(60.9)	34(49.3)	35(50.7)	28(40.6)	41(59.4 )
	6	215	67.0	98(45.6)	117(54.4)	102(47.4)	113(52.6)	106(49.3)	109(50.7)
	8	24	7.5	14(58)	10(41.7)	11(45.8)	13(54.2)	11(45.8)	13(54.2)
	Other	13	4.0	7(53.8)	6(46.2)	3(23.0)	10(77.0)	11(84.6)	2(15.4)
								p = 0.034^†^	
	OR^||^							0.499 (0.303-0.754)	
**Length of experience in Nursing (years)**	< 3	87	27.1	41(47.1)	46(51.9)	44(50.6)	43(49.4)	48(55.1)	39(44.9)
	3 to 10	126	39.3	60(47.6)	66 (52.4)	61(48.4)	65(51.6)	59(46.8)	67(53.2)
	> 10	108	33.6	45(41.7)	63(58.3)	45(42)	63(58.3)	49(45.4)	59(54.6)
**Work shift**	Daytime	222	69.2	104(46.9)	118(53.1)	104(46.9)	118(53)	113(50.9)	109(49.1)
	Night	69	21.5	25(36.2)	44(63.8)	35(50.7)	34(49.3)	28(40.6)	41(59.4)
	Mixed^¶^	30	9.3	17(56.7)	13(43.3)	11(36.7)	19(63.3)	15(50.0)	15(50.0)
**Weekly workload (hours)**	30/36	271	84.4	124(45.7)	147(54.3)	124(45.7)	147(54.3)	130(48.0)	141(52.0)
	40/44	43	13.4	20(46, 5)	23(53.5)	23(53.5)	20(46.5)	21(48.8)	22(51.2)
	Other	7	2.2	2(28.6)	5(71.4)	3 (42.8)	4(57.2)	5(71.4)	2(28.6)
**Time since graduation (years)**	< 5	119	37.1	56(47.0)	63(53.0)	53(44)	66(55.5)	64(53.8)	55(46.2)
	6 to 10	97	30.2	46(47.4)	51(52.6)	54(55.7)	43(44, 3)	43(44.3)	54(55.7)
	> 10	105	32.7	44(41.9)	61(58.1)	43(41.0)	62(59.0)	49(46.6)	56(53.4)
Unit in which it operates^**^	Critical	162	50.5	72(44.4)	90(55.6)	75(46.3)	87(53.7)	74(45.7)	88(54, 3)
	Assistance	116	36.1	48(41.4)	68(58.6)	60(51.7)	56(48.3)	63(54.3)	53(45.7)
	Administrative	43	13.4	26( 60.5)	17(39.5)	15(34.9)	28(65.1)	19(44.2)	24(55.8)
**Graduate**	Yes	82	25.5	32(39.0)	50( 61.0)	28(34.1)	54(65.9)	24(29.3)	58(70.7)
	No	239	74.5	114(47.7)	125(52.3)	122(51.0)	117(49.0)	132(55.2)	107(44.8)
						p = 0.006^§^		p<0.001^§^	
	OR					0.497 (0.295-0.838)		0.335 (0.196-0.575)	
	Linear regression					[F1.390 = 7.11, p=0.008; R^2^=0.022		[F1,390 = 17.25, p=0.000; R^2^=0.051	
**Has another employment relationship**	Yes	60	18.7	24(40.0)	36(60.0)	26(43.3)	34(56.7)	23(38.3)	37(61.7)
	No	261	81.3	122(46.7)	139(53.3)	124(47.5)	137(52.5)	133(51.0)	128(49.0)
**Total**		321	100	146(45.5)	175 (54.5)	150(46.7)	171(53.3)	156(48.6)	165(51.4)

*JSS = Job Stress Scale; ^†^Chi-square test significant for p < 0.05; ^‡^For the calculation of the odds ratio (OR) of age, two categories were considered: older than or equal to 40 years or younger than 40 years; ^§^Fisher’s exact test significant for p < 0.05; ^||^For the calculation of OR of the workday, six hours/day *versus* other workloads were considered; ^¶^Mixed: day or night shift, swapping time off; ^**^Critical (Intensive Care Units, Emergency, Surgical Center, Maternity, Obstetric Center, Hemodialysis and Oncology Center); Care (Inpatient Units and Heart Institute) and Administrative (outpatient and specialties, diagnostic support services and administrative area)

### Ethical aspects

All ethical precepts were observed as recommended in Resolutions 466/2012 and 510/2016^20^, of the National Health Council (NHC) on research with human beings. After the hospital’s authorization, the study was submitted to the University’s Research Ethics Committee under CAAE No. 18791319.7.0000.5350 and approved under Opinion No. 3.657.852. 

## Results

A total of 321 Nursing professionals participated in the research. Of these, 83 (25.9%) were nurses and 238 (74.1%) Nursing technicians. The sample was predominantly female (90%), aged up to 40 years (75.7%) and married (59.5%). Regarding the work characteristics of the participants, 86.6% worked in direct patient care units, 69.2% were allocated during the daytime, 67.0% had a six-hour shift and 84.4% had 30/36-hours work a week. Furthermore, 81.3% stated that they had only one employment relationship.

Table 1 presents the socio-demographic and work characteristics according to the JSS dimensions. The hypothesis of independence between psychological demands and professional category was rejected (p = 0.009), with a higher proportion of high demand among nurses; between high control over work and age (p = 0.044), with better results among those aged over 40 years, with age representing a risk factor for exposure to occupational stress. Other variables, besides being statistically associated, represented a protection factor and are related to lower levels of stress. They are: professional category/position as a nurse (p<0.001), managerial position (p = 0.009) and post-graduation course (p = 0.006) and between high social support and professional category/position as a nurse (p<0.001), managerial position (p = 0.033), 12-hour working day (p = 0.034) and post-graduation course (p<0.001). The simple linear regression showed that control over work has changed in individuals older than 40 years (F1.390 = 5.59, p=0.019; R^2^=0.017).

**Figure 1:**
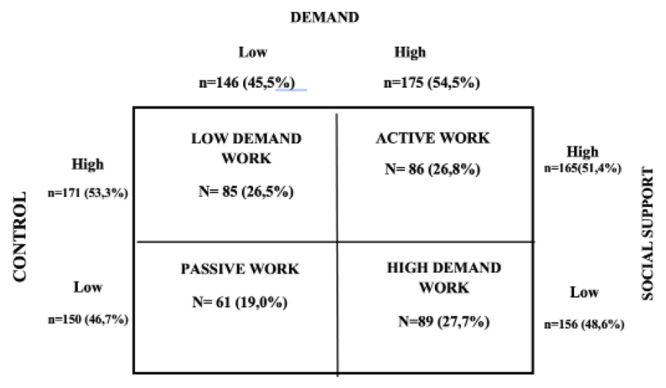
Distribution of Nursing professionals (n = 321) according to the quadrants of the DCM, according to the dichotomization of the Job Stress Scale (JSS) dimensions. Ijuí, RS, Brazil, 2019-2020^15^ , ^21^

Figure 1 presents the distribution of Nursing professionals according to the stratification in the quadrants of the DCM from the dichotomization of the three dimensions proposed by the JSS: demand, control and social support. It was found that the participants’ work is characterized by high psychological demand (54.5%), high control (53.3%) and there is perception of high social support (51.4%). In the combination of the quadrants of the DCM, 89 (27.7%) were in high demand work, 86 (26.8%) in active work, 85 (26.5%) in low demand work, and 61 (19.0%) in passive work.

Table 2 presents the results regarding the frequency of resilience of the Nursing professionals participating in the study according to the JSS dimensions. It is evident that, of these, 175 (54.5%) had moderate resilience and 117 (36.4%), high resilience. Although there was no statistically significant association between resilience and the JSS dimensions, it was observed that a higher percentage of professionals presented moderate resilience, with a higher frequency of low psychological demand, low control at work, and high social support. Among the professionals with high resilience, there was a higher frequency of high psychological demand and high control at work and high social support.

**Table 2:** Frequency of resilience of Nursing professionals (n = 321) according to the dimensions of the JSS^*^. Ijuí, RS, Brazil, 2019-2020

Dimensions of the JSS		Resilience				p-value^†^
		Low	Average	High	Total	
		n (%)	n (%)	n (%)	n (%)	
**Demand**	Low	12(8.2)	87(59.6)	47(32.2)	146(45.5)	0.25
	High	17(9.7)	88(50.3)	70(40.0)	175(54.5)	
**Control**	Low	19(12.6)	82(54.7)	49(32.7)	150(46.7)	0.07
	High	10(5.8)	93(54.4)	68(39.8)	171(53.3)	
**Social support**	Low	11(7.0)	79(50.7)	66(42.3)	156(48.6)	0.08
	High	18(10.9)	96(58.2)	51(30.9)	165(51.4)	
**Total**		29(9.1)	175(54.5)	117(36.4)	321(100)	

*JSS = Job Stres Scale; ^†^Chi-square test, significant for p < 0.05

Table 3 shows the averages of the RS factors according to each JSS dimension. There was a statistically significant difference between Factor I of resilience - resolutions of actions and values that give meaning to life - and control over work (p = 0.04), with higher average among those with high control, and between Factor I of resilience and social support (p = 0.002), with higher average among those who perceived low social support.

**Table 3:** Resilience of Nursing professionals (n=321) according to the dimensions of the JSS*. Ijuí, RS, Brazil, 2019/2020

Dimensions of the JSS	Resilience^†^	Descriptive statistics						p-value^¶^
		n	Li^‡^	Ls^§^	Mean	SD^||^	Median	
**Demand**	Factor I							
	Low	146	23	91	77.33	8.48	78	0.46
	High	175	19	91	76.60	11.87	79	
	Factor II							
	Low	146	16	38	28.23	4.57	29	0.11
	High	175	11	40	28, 87	5.32	30	
	Factor III							
	Low	146	9	35	28.71	3.83	29	0.09
	High	175	8	35	29.08	4.57	30	
**Control**	Factor I							
	Low	150	23	91	75.84	10.72	77.5	0, 04
	High	171	19	91	77.89	10.16	79	
	Factor II							
	Low	150	11	40	28.21	5.02	29	0.30
	High	171	11	39	28.90	4.97	29	
	Factor III							
	Low	150	8	35	28.82	4, 50	29	0.95
	High	171	8	35	28.99	4.03	29	
**Social support**	Factor I							
	Low	156	19	91	78.15	10.94	79	0.002
	High	165	24	91	75.78	9.88	78	
	Factor II							
	Low	156	12	39	28.76	4.87	30	0.41
	High	165	11	40	28.40	5.12	29	
	Factor III							
	Low	156	9	35	28.94	4.50	29.5	0.43
	High	165	8	35	28.88	4.01	29	

*JSS = Job Stres Scale; ^†^Resilience: Factor I = Resolutions of actions and values; Factor II = Independence and determination; Factor III = Self-confidence and ability to adapt to situations; ^‡^Li = Lower limit; ^§^Ls = Upper limit; ^||^DP = Standard deviation; ^¶^Mann-Whitney U test, significant for p < 0.05

The association between the resilience of the participants and exposure to occupational stress is presented in Table 4. It is found that, of the Nursing professionals who were in the quadrant of high-demand work - higher health risk, 53.9% had moderate resilience and 34.0%, high. However, there was no statistically significant difference between the quadrants of the DCM and the resilience.

**Table 4:** Occupational stress exposure and resilience of Nursing professionals (n = 321). Ijuí, RS, Brazil, 2019-2020

Quadrants MDC	Resilience				p-value*
	Low	Moderate	High	Total	
	n (%)	n (%)	n (%)	n (%)	
High-demand	9(10, 1)	48(53.9)	32(34.0)	89(27.7)	0.49
work Low-demand	2(2.3)	53(62.3)	30(35.3)	85(26.5)	
work Passive	10(16)	34(55.7)	17(27.9)	61(19.0)	
work Active work	8(9.3)	40(46.5)	38(44.2)	86(6.8)	
Total	29(9, 0)	175(54.5)	117(36.4)	321(100)	

*Chi-square test

## Discussion

Personal and organizational characteristics of Nursing work in the hospital environment contribute to professional illness, while they may be associated with lower risk of exposure to occupational stress. This statement emerges from reflections based on the results of this study, which demonstrated that a higher percentage of Nursing professionals who worked in a general hospital presented moderate and high resilience. They perceived high control over their work and high social support, which, associated with resilience, can contribute to the reduction of exposure to occupational stress.

Unsatisfactory working conditions, organizational conflicts, lack of control over results, increased clinical severity and patient expectations, helplessness in the face of death and relational difficulties with family members are among the multitude of factors that can negatively impact the health of Nursing professionals^22^. Occupational stress arises when the worker exceeds his individual and social capacity to cope with the psychological demands and difficulties experienced in the work environment^23^.

The fact that 73.5% of the Nursing professionals participating in this study presented some degree of exposure to stress and that 27.7% of them were in the quadrant of highly demanding work is worthy of attention, since this situation can have negative repercussions on the work environment. There was the perception, by the worker, that the high social support and the control over the work performed are protective factors against stress exposure^2^. Support from colleagues and supervisors in performing tasks, social integration and a trusting relationship in the group contribute to the prevention of the harmful effects of work-related stress on the worker’s health^21^. 

Another result indicating an alert exposure to stress is the sum of the percentage of workers who performed passive work (19.0%) to the percentage of those who were in highly demanding work (27.7%), which shows that almost half of the participants were in the health risk quadrants. Passive work leads the worker to loss of skills and disinterest in work^24^. High-demand activities, on the other hand, are considered harmful to health, since high stress can manifest itself in fatigue, depression, physical and cardiovascular symptoms, and anxiety^25^. 

Nursing care in the hospital environment requires from the professional expertise, constant attention, agility, decision making and concomitant execution of several tasks, among other particularities that result in high psychological demands. The complexity of care and the care of all basic human needs that involve, including, emotional support and guidance to the patient, extensive to his/her family, when associated with physical exhaustion, demand significant effort from the professionals to face the difficulties and to prevent labour illness^23^.

The results of this research, regarding the association between exposure to stress and personal and labour characteristics, lead to reflections on how stress can interfere in the personal, professional and institutional daily lives of Nursing workers. The analysis of the JSS dimensions in relation to exposure to stress, when divided by professional category, shows that nurses presented a higher proportion of high psychological demand in comparison to Nursing technicians. However, nurses perceived high control over work and high social support, while among technicians, a higher percentage stated high psychological demand, low control and low social support, which refers to the probability of belonging to the quadrant of highly demanding work.

This result may be influenced by the organizational structure of the work of Nursing, considering that it is the exclusive responsibility of nurses to plan, manage, coordinate, prescribe and evaluate Nursing care, which involves not only the care itself, but also the management of personnel, materials, equipment and structure necessary for care^26^. At the same time, this study instigates new investigations, even with other methodological designs, of how much the dimensioning of the Nursing staff, both quantitative and qualitative, can influence the results.

Also in the relationship between socio-demographic and labour characteristics and exposure to stress, the results show that expertise in the area of work, age over 40 years and management positions were associated with a perception of greater control over work, which favours greater professional autonomy. In the same way, professional expertise, management positions and a 12-hour work day favored the perception of greater social support to the worker. The team decision-making process and the support among professionals favour the working conditions^27^. Insofar as the relationship with the other reflects the worker’s own weaknesses and potentialities, teamwork contributes to a resilient *praxis* ^27^.

A systematic review that aimed to identify the main psychosocial factors in Nursing work indicated that the perception of justice, respect, support from supervisors and social inclusion favour the preservation of the mental health of Nursing workers^5^. The subjective and individual character in the perception of factors that contribute to stress and the need for health-promoting interventions focused on psychosocial characteristics, which enable the active participation of professionals, are highlighted^28^.

In this sense, the results of this research show that, together with the socio-demographic and labour characteristics that favour the prevention of illness, resilience in coping with work stress was used by Nursing professionals. This statement can be justified by the fact that 91% of the participants presented moderate and high resilience. Furthermore, the relationship of resilience with higher averages of high control over work and low social support indicates that these professionals used resilience to solve actions and to defined values that give meaning to life and to work itself. Resilience involves independence, power and decisions of the individual to plan and solve problems^29^. Problem-centered problem solving is considered a cognitive strategy in which the individual recognizes adversity and seeks alternative solutions focusing on the positive aspects involved^3^. 

The sum of the percentage of professionals in active work (26.8%) to those who were in a low demand job (26.5%) showed that a little more than half of the participants were in a range considered to be at a lower risk of getting sick. Increased control over work is associated with better health assessment and lower levels of stress^25^. Active work is when high demands and high control of work coexist, which enables the worker to learn, to grow personally and to plan strategies to better cope with stress. 

Finally, despite a not worrisome figure in terms of occupational health, it is noteworthy that 9% of the participants in this study showed low resilience. Resilience is a competence that can be developed^17^. Facing the difficulties experienced by Nursing professionals requires individual and institutional actions, strategies and interventions that favour the promotion and expansion of resilience as a positive force for overcoming adversity^29^. 

The analysis of the results of this research shows the relevance of implementing individual, collective, and management strategies to maintain and increase the resilience of Nursing professionals. It also highlights the importance of self-care and constant evaluation of determining and conditioning factors for the health of the worker.

The results of this study are important because they provide an opportunity and subsidize reflections about the work of Nursing, the risk of exposure to occupational stress and the importance of resilience for the prevention of occupational disease. The data can be useful for Nursing professionals and managers in the planning, implementation and management of actions to promote occupational health. In the same way, the results presented here can alert, encourage and subsidize regulatory and representative entities of Nursing to institute local, state and national measures as guidelines to ensure adequate and favourable working conditions for professional practice.

However, among the limitations of this investigation, the fact that it was conducted in only one institution limits the possibility of generalizing and comparing the results due to the peculiarities of each institution and the bio-psychosocial and occupational factors. 

## Conclusion

The analysis of the association between resilience and occupational stress shows that Nursing professionals who worked in a general hospital had moderate and high resilience. And that resilience, high control over work and high social support may contribute to the reduction of exposure to occupational stress. Personal, professional and work characteristics, such as age over 40 years, represented a risk factor for exposure to stress. On the other hand, working as a nurse, holding a management position, working 12-hour shifts and taking a graduate course are protective factors and are associated with lower levels of stress. Therefore, in this population, it was not possible to conclude that resilience contributed to the reduction of occupational stress.

A greater contribution of knowledge about occupational health and promotional and preventive actions to these workers is essential, especially to strengthen social support in the work of Nursing technicians. Detailed studies are also needed, including other methodological designs, on individual and organizational factors, as well as interventions to reduce the negative impacts on the health of workers and the safety of patients, professionals and the institution.
